# Intelligent Transportation Related Complex Systems and Sensors

**DOI:** 10.3390/s21062235

**Published:** 2021-03-23

**Authors:** Kyandoghere Kyamakya, Jean Chamberlain Chedjou, Fadi Al-Machot, Ahmad Haj Mosa, Antoine Bagula

**Affiliations:** 1 Institute for Smart Systems Technologies, Universität Klagenfurt, A9020 Klagenfurt, Austria; Jean.Chedjou@aau.at (J.C.C.); ahmad.haj.mosa@pwc.com (A.H.M.); 2 Department of Applied Informatics, Universität Klagenfurt, A9020 Klagenfurt, Austria; Fadi.AlMachot@aau.at; 3 ISAT Laboratory, University of the Western Cape, Bellville 7535, South Africa; abagula@uwc.ac.za

Building around innovative services related to different modes of transport and traffic management, intelligent transport systems (ITSs) are being widely adopted worldwide to improve the efficiency and safety of the transportation system. They enable users to be better informed and make safer, more coordinated, and smarter decisions on the use of transport networks. Current ITSs are complex systems, made up of several components/sub-systems characterized by time-dependent interactions among themselves. Some examples of these transportation-related complex systems include road traffic sensors, autonomous/automated cars, smart cities, smart sensors, virtual sensors, traffic control systems, smart roads, logistics systems, smart mobility systems, and many others that are emerging from niche areas. The efficient operation of these complex systems require (i) efficient solutions to the issues of sensors/actuators used to capture and control the physical parameters of these systems as well as the quality of data collected from these systems; (ii) tackling complexities using simulations and analytical modelling techniques; (iii) applying optimization techniques to improve the performance of these systems. 

This book out of the Special Issue on Intelligent Transportation Related Complex Systems and Sensors emerges as a result of the crucial need for improving transportation support in different domains and parts of the world by finding solutions to the rich yet non-trial and unexpected behaviour resulting from the complexity of ITS. It includes twenty-four papers, which cover scientific concepts, frameworks, architectures, and various other ideas on analytics, trends, and applications of transportation related data. 

The 24 papers/chapters contained in this book propose solutions to various issues and broadly grouped into four classes/parts, namely, the following ones:

1. Traffic safety and security.
2. Autonomy and path planning.
3. Traffic density.
4. Traffic analytics and prediction.

## 1. Safety and Security

In [1], the authors considered the research gap found in the observability in traffic networks. The work addressed non-definitive plate scanning problems, by using sensors embedded into elements across traffic network to enable technicians reach better conclusions when they deal with traffic network analysis. This is an area of research with very limited number of studies, and the authors in this work proposed (i) an architecture for deploying temporary low-cost sensors across city streets as an alternative of rubber hoses commonly used in elaborate urban mobility plans; (ii) a design for these low-cost, low-energy sensors themselves; (iii) an ideal sensor location model for establishing the best set of network links to achieve the study’s aims. To demonstrate the viability of these contributions, a case study with the installation of a set of proposed devices is used by the authors to demonstrate the viability of their contributions.

In [2], the effects of drivers’ mental state and passenger compartment conditions on driving performance and driving stress were considered. Factors such as the human error, cognitive capacity, and levels of CO_2_ concentration, humidity, and temperature within the vehicles were analysed in terms of their impact on driving. The experimental setting of the study included a survey with 50 drivers, 25 min of drive time using a driving simulator. Information about the drivers’ mental state and stress levels were monitored during the test using biometric sensors, while suitable sensors were used to monitor environmental conditions—temperature, humidity, and CO_2_ levels. The study revealed that i) the initial level of stress and tiredness of the driver can have a strong impact on stress, driving behaviour, and fatigue and ii) elements such as state of the mind (sadness or happiness) and the conditions of the interior of the vehicle can also impaired driving and affect compliance with traffic regulations. 

Reference [3] addressed the issues associated with expert assessments and statistical studies commonly used to evaluate the impact of intelligent transport system (ITS) services on road safety. The work built upon an approach based on surrogate safety measures calculated and calibrated with the use of simulation techniques and a driving simulator to achieve traffic efficiency and road safety. Experiments were conducted to measure the influence of selected scenarios of variable speed limits on the efficiency and safety of traffic on sections of motorways and expressways in various traffic conditions. The presented studies confirmed the positive impact of variable speed limits (VSLs) on the level of road safety and traffic efficiency. Recommendations were then given as well as areas of potential further research.

Building upon an intelligent traffic control system installed in Poland, [4] addressed the issue of safety and traffic operation on roads by (i) analysing the safety level of the entire road network when traffic is rerouted to paths along different road categories, intersections, road environments, and densities of access points and (ii) presenting a comparison between traffic operation and road safety performance, with the aim of predicting the number of crashes for each possible route when considering travel time and delay. The results of the study allow for maximizing safety or traffic operation characteristics, providing an effective tool for the management of rural road systems. 

In [5], a review of data analytic applications in road traffic safety was done. The aim was to reduce the start-up burden of data collection and descriptive analytics for statistical modelling and route optimization of risk associated with motor vehicles. Building upon a data-driven bibliometric analysis, the study showed that literature is divided into two disparate research streams: (a) predictive or explanatory models that attempt to understand and quantify crash risk based on different driving conditions and (b) optimization techniques that focus on minimizing crash risk through route/path-selection and rest-break scheduling. Translation of research outcomes between these two streams was limited. The study also (i) presented publicly available high-quality data sources (different study designs, outcome variables, and predictor variables) and descriptive analytic techniques (data summarization, visualization, and dimension reduction) that could be used to achieve safer-routing and provide code to facilitate data collection/exploration by practitioners/researchers; (ii) reviewed the statistical and machine learning models used for crash risk modelling; (iii) showed that (near) real-time crash risks are rarely considered. 

Reference [6] also reviewed data analytic applications in road traffic safety with the objective of filing the gap left by [5] between the predictive and prescriptive models pertaining to crash risk prediction and minimization, respectively. The paper reviewed and categorized the optimization and prescriptive analytic models that focused on minimizing crash risk. The review showed that, though majority of works in this segment of the literature are related to the hazardous materials (hazmat) trucking problems, with some exceptions, many studies can also be utilized in non-hazmat scenarios. In an effort to highlight the effect of crash risk prediction models on the accumulated risk obtained from the prescriptive model, the review presented a simulated example where four risk indicators (obtained from logistic regression, Poisson regression, XGBoost, and neural network) were used in the *k*-shortest path algorithm. An example demonstrating two major designed takeaways were also presented in the paper. The first revealed that the shortest path may not always result in the lowest crash risk, while the other showed that a similarity in overall predictive performance may not always translate to similar outcomes from the prescriptive models. 

With regards to intrusion detection, the work done in [7] revisited the issue of labelling of alarm regions in video surveillance-based intrusion detection systems. The authors proposed a three step adaptive segmentation algorithm to delineate the boundary of the track area with very light computation burden. In the first step of the algorithm, the image was segmented into fragmented regions. During this step, an optimal set of Gaussian kernels with adaptive directions for each specific scene was calculated using Hough transformation to reduce the redundant calculation in the evaluation of the boundary weight for generating the fragmented regions. At the second step of the algorithm, the fragmented regions were combined into local areas using a new clustering rule based on the region’s boundary weight and size. Lastly, a classification network is used to recognize the tracked area among all local areas. To achieve fast and accurate classification, a simplified convoluted neural network (based on pre-trained convolution kernels) and a loss function (that can enhance the diversity of the feature maps) were used. Obtained results showed that the proposed method found an effective balance between the segmentation precision, calculation time, and hardware cost of the system. 

On a related issue of traffic videos, [8] sought to tackle the issue of restoring traffic videos with different degrees of haziness in a real-time and adaptive manner. They work proposed an efficient traffic video dehazing method using adaptive dark channel prior and spatial–temporal correlations. The work used a haziness flag to measure the degree of haziness in images based on dark channel prior. It then got the adaptive initial transmission value by establishing the relationship between the image contrast and haziness flag. Additionally, the method took advantage of the spatial and temporal correlations among traffic videos to speed up the dehazing process and optimize the block structure of restored videos. Extensive experimental results showed that the proposed method had superior haze removing and colour balancing capabilities for the images with different degrees of haze and was indeed able to restore degraded videos in real time. 

## 2. Autonomy and Path Planning

The development of human-like autonomous driving systems has in recent times gained increased attention from both technology companies and academic institutions alike, as it has the potential to improve the interpretability and acceptance of autonomous vehicle systems. [9] addressed the research gap found in the planning of a safe and human-like obstacle avoidance trajectory, which is one of the critical issues for the development of autonomous vehicles (AVs). The paper proposed an automatic obstacle avoidance system that focused on the obstacle avoidance characteristics of human drivers. Different models for trajectory planning and trajectory tracking were proposed and tested through off-line simulation based on CarSim/Simulink. Simulation results revealed that the proposed trajectory planning and tracking controllers were more human-like under the premise of ensuring the safety and comfort of the obstacle avoidance operation, thus providing a foundation for the development of AVs. 

The work done in [10] relied on the license plate data obtained from the massive volume of information collected from video imaging to predict vehicle trajectory. The paper proposed a real-time vehicle trajectory prediction method based on (i) historical trip rules extracted from vehicle license plate data in an urban road environment; (ii) vehicle trip chain acquired on the basis of the topologic graph of the road network, channelization of intersections, and the driving status information at intersections; (iii) a trip chain compensation method based on the Dijkstra algorithm to complement missing data in the original vehicle license plate. The proposed method was tested using realistic road traffic scenarios with actual vehicle license plate data. Good trajectory prediction results were obtained and revealed an average accuracy of 0.72 for one-step prediction when there are only 200 historical training data samples. 

In [11], the authors studied the effect of travel time information on day-to-day driver route choice behaviour. A real-world experimental study designed to have participants repeatedly choose between two alternative routes for five origin–destination pairs over multiple days was performed. The participants were providing with dynamically updated travel time information (average travel time and travel time variability) during the experiment. The results of the study revealed that (i) historical travel time information enhanced behavioural rationality by 10% on the average; (ii) expected travel time information was more effective than travel time variability information in enhancing rational behaviour when drivers had limited experiences; (iii) when drivers lack experience, the faster less reliable route was more attractive than the slower more reliable route; (iv) with cumulative experiences, drivers become more willing to take the more reliable route given that they are reluctant to become risk seekers once experience is gained; (v) the effect of information on driver behaviour differed significantly by participant and trip, which to a large extent, depended on personal traits and trip characteristics.

## 3. Traffic Density

References [12,13,14] focused on vehicular and/or human traffic. In [12], the authors estimated traffic stream density by using data from solely connected vehicle (CV) and applying a nonlinear filtration. A particle filter (PF) was developed to produce reliable traffic density estimates using the CV’s travel-time measurements. Traffic flow continuity was then used to derive the state equation, while the measurement equation was derived from the hydrodynamic traffic flow relationship. A comparison was done against two PF filtering approach, namely Kalman filter (KF) and adaptive KF (AKF). Obtained results revealed that (i) the three techniques produce accurate estimates—with the KF, surprisingly, being the most accurate of the three techniques (ii) the accuracy of the PF estimations increased as the number of particles increased and (iii) the accuracy of the density estimate increased as the level of CV market penetration increased. 

In a similar work, [13] used the adaptive Kalman filter (AKF) to reliably estimate traffic vehicle count by considering real-time characteristics of system noise. Using only real-time probe vehicle data, the AKF is demonstrated to outperform the traditional Kalman filter by reducing the prediction error by up to 29%. A novel approach was also introduced by the paper wherein AKF was combined with a neural network (AKFNN) to enhance the vehicle count estimates. The results showed that the accuracy of vehicle count estimates was significantly improved when AKFNN was used by up to 26% compared to pure AKF, but the AKF was more sensitive to the initial conditions.

Reference [14] slightly shifted focus to passenger count in rail transportation. The work revisited the issue of forecasting passenger flows on metro lines by proposing the use of artificial neural networks (ANNs). The authors forecasted the number of passenger flows on a metro to be a function of passenger counts at station turnstiles. The study assumes that metro station turnstiles record the number of passengers entering by means of an automatic counting system and that these data are available every few minutes. These data are then used to estimate the onboard passengers on each track section of the line (i.e., between two successive stations). The ANNs were trained using simulation data obtained with a dynamic loading procedure of the rail line and tested using real-scale case scenario of Line 1 of the Naples metro system in Italy. The numerical results showed that the proposed approach was able to forecast the flows on metro sections with satisfactory levels of precision.

## 4. Analytics and Prediction

These papers focus on analysis of transportation data for the purpose of drawing insights or predicting future events. 

Reference [15] addresses the requirement of high level of detail and coverage in traffic data acquisition in the next generation of intelligent transportation systems (ITS). The authors presented appropriate methods with consideration of realistic scale and accuracy conditions of the original data acquisition. The study relied on datasets consisting of timestamp and speed for each individual vehicle used as input data and proposed as a first step, a closed formulation for a sensor offset estimation algorithm with simultaneous vehicle registration. Building upon this initial step, the datasets are fused to reconstruct microscopic traffic data using quintic Beziér curves. The derived trajectories were then used to thoroughly investigate the dependency of the results on the accuracy of the individual sensors. It was found that the proposed method enhanced the usability of common cross-section-based sensors by enabling the deriving of non-linear vehicle trajectories without the necessity of precise prior synchronization. 

The focus on [16] lies on the challenging issue of accurate modelling of short-term traffic prediction due to its intricate characteristics, stochastic, and dynamic traffic processes. Existing works in this area followed different modelling approaches focusing on speed, density, or data volume. The study however used (i) state-of-the-art models via hyper-parameter optimization using different machine learning classifiers such as local deep support vector machine (LD-SVM), decision jungles (DJ), multi-layers perceptron (MLP), and CN2 rule induction and (ii) traffic states evaluation based on traffic attributes such as level of service (LOS) horizons and simple if–then rules at different time intervals. The findings of the study revealed that (i) hyper-parameter optimization via random sweep yielded superior results; (ii) the overall prediction performances obtained an average improvement by over 95%, such that the decision jungle and LD-SVM achieved an accuracy of 98.2 and 97.5%, respectively; (iii) the robustness and superior performances of decision jungles (DJ) over other methods.

Still on short distance traffic, [17] proposed a solution to the issue of bicycle sharing systems (BSSs), which are traditionally conceived as a last-mile complement to the public transport system. The paper demonstrated that BSSs can be seen as a public transport system in their own right and built a mathematical framework for the classification of BSS trips. It also used trajectory information to create the trip index, which characterizes the intrinsic purpose of the use of BSS as transport or leisure. By applying the proposed methodology to empirical data from BiciMAD (the public BSS in Madrid, Spain), the authors conducted experiments, which revealed that the obtained trip index was able to correctly distinguish between transport and leisure categories using exhibited statistical and operational features. 

In identifying irregular/abnormal road surface conditions, the authors in [18] proposed an efficient and low-cost model, which used the vibration and acceleration sensors in smartphones. The study used an improved Gaussian background model to extract the features of the abnormal pavement and the k-nearest neighbour (kNN) algorithm to distinguish the abnormal pavement types, including pothole and bump. The study also included as feature the influence of vehicles with different suspension characteristics on the detection threshold and proposed an adaptive adjustment mechanism based on vehicle speed. In determining the accuracy of the proposed model, the authors bench-marked their algorithms against real-life field investigation. Obtained results showed that the vibration and acceleration information could indeed reveal the condition of the road surface with an accuracy of up to 96.03% for road surface pothole and 94.12% for road surface bumps. These results show that the proposed road surface recognition method could potentially be utilized to replace special patrol vehicles for timely and low-cost road maintenance. 

The study in [19] revisits the costly and time-consuming issue of subjective evaluation of vehicle ride comfort for vehicle development. In contrast to most of the approaches that rely on the use of a regression model between objective metrics and subjective ratings with an accuracy that is highly dependent on the selection of the objective metrics, the solution proposed in the study used a method that built a correlation model between measurements and subjective evaluations without using predefined features or objective metrics. Using a combination of (i) numerical representation of ride comfort extracted from raw signals based on the idea of the artistic style transfer method, (ii) a correlation model designed based on the extracted numerical representation and subjective ratings, and (iii) a pre-trained neural network, the proposed model was proven to provide much better accuracy than any other correlation models in the literature. 

Focusing on vehicle interior noise classification, the study in [20] developed a multi-classification model that has the potential to be used to analyse the causes of abnormal noise using statistical methods and evaluate the effect of rail maintenance work. The work first developed a multi-source data (audio, acceleration, and angle rate) collection framework via built-in sensors in smartphone. It then used the Shannon entropy based on a 1-second window to segment the time-series signals. Forty-five features extracted from the time and frequency domains were used to establish the classifier. The study investigated the effects of balancing the training dataset with the synthetic minority oversampling technique (SMOTE). It then compared and analysed the classification results of importance-based and mutual information-based feature selection methods using a feature set consisting of the top 10 features by importance score. Comparisons with other classifiers indicated that the proposed XGBoost-based classifier ran fast, while maintaining good accuracy. 

Reference [21] aimed at bridging the research gap found in the field of rail surface scratching data, where only a limited number of studies have addressed the issue of complete closed mesh model. The paper proposed a model based on a novel triangulation algorithm relying on the topological features of the point-cloud model (PCM) of scratch data. These data were obtained by implementing a scratch-data-computation process following a rail-geometric-feature-fused algorithm of random sample consensus (RANSAC) constructed by 3D laser vision. Using a method that is universal for all types of normal-speed rails in China, the paper presented experimental results showing that the proposed method could accurately acquire the complete closed mesh models of scratch data of one meter of 50 Kg/m-rails within 1 min. 

In contrast to the intrusive type cylinder pressure sensor, which has high cost, low reliability, and short life due to severe working environments, [22] proposed the cylinder pressure identification method also called virtual cylinder pressure sensor. In this work, frequency–amplitude modulated Fourier series (FAMFS) and extended-Kalman-filter-optimized (EKF) engine model were used as low-cost, real-time, non-invasive, and highly accuracy alternative solutions. The paper established an iterative speed model based on burning theory and law of energy conservation and used the efficiency coefficient to represent the operating state of engine from fuel to motion. The iterative speed model was associated with the throttle opening value and the crankshaft load. EKF was used to estimate the optimal output of this iteration model, which was utilized to separately compute the frequency and amplitude of the cylinder pressure cycle-to-cycle. A standard engine’s working cycle, identified by the 24th order Fourier series was then determined. Using frequency and amplitude obtained from the iteration model to modulate the Fourier series yielded a complete pressure model. Using a commercial engine (EA211) provided by the China FAW Group corporate R&D centre, the proposed method was verified through a test that revealed its high accuracy and real-time capability, with an error percentage for speed of less than 9.6% and the cumulative error percentage of cylinder pressure less than 1.8% when A/F Ratio coefficient is setup at 0.85. 

Building upon game theory, [23] presented a novel de-centralized flexible phasing scheme, cycle-free, adaptive traffic signal controller that uses a Nash bargaining game-theoretic framework. The Nash bargaining algorithm was used to optimize the traffic signal timings at each signalized intersection by modelling each phase as a player in a game, where players cooperate to reach a mutually agreeable outcome. The controller is implemented and tested in the integration microscopic traffic assignment and simulation software. Its performance was then compared with traditional decentralized adaptive cycle length and phase split traffic signal controller, a centralized, fully coordinated, adaptive phase split, cycle length, and offset optimization controller. Using comparisons conducted in the town of Blacksburg, Virginia (38 traffic signalized intersections) and in downtown Los Angeles, California (457 signalized intersections), the results showed significant potential benefits of using the proposed controller over other state-of-the-art centralized and de-centralized adaptive traffic signal controllers on large-scale networks both during uncongested and congested conditions.

Building upon dynamical methods to realize a time-varying matrix inversion, [24] relies on a system of coupled ordinary differential equations (ODEs), constituting a recurrent neural network (RNN) model as a universal modelling framework for realizing a matrix inversion, provided the matrix is invertible. The study in this paper builds around this framework to propose and investigate a new combined/extended method for time-varying matrix inversion that extends both the gradient neural network (GNN) and the Zhang neural network (ZNN) concepts. The newly proposed model has (i) proven that it has exponential stability according to Lyapunov theory, (ii) a much better theoretical convergence speed compared to other previous related methods (namely GNN, ZNN, Chen neural network, and integration-enhanced Zhang neural network or IEZNN), and (iii) a better practical convergence rate when compared to the other models on practical examples and both their respective measured convergence and error rates.
